# Effects of Moderate Alcohol Consumption in Non-Alcoholic Fatty Liver Disease

**DOI:** 10.3390/jcm11030890

**Published:** 2022-02-08

**Authors:** Peter Lemmer, Paul Manka, Jan Best, Alisan Kahraman, Julia Kälsch, Ramiro Vilchez-Vargas, Alexander Link, Hsin Chiang, Guido Gerken, Ali Canbay, Lars P. Bechmann, Svenja Sydor

**Affiliations:** 1 Department of Internal Medicine, University Hospital Knappschaftskrankenhaus, Ruhr-University Bochum, 44892 Bochum, Germany; peter.lemmer@gmx.net (P.L.); paul.manka@rub.de (P.M.); jan.best@kk-bochum.de (J.B.); ali.canbay@rub.de (A.C.); lars.bechmann@rub.de (L.P.B.); 2 Department of Gastroenterology, Hepatology and Infectious Diseases, Otto-von-Guericke-University Magdeburg, 39120 Magdeburg, Germany; ramiro.vilchez@med.ovgu.de (R.V.-V.); Alexander.Link@med.ovgu.de (A.L.); 3 Department of Gastroenterology and Hepatology, University Hospital, University Duisburg-Essen, 45147 Essen, Germany; alisan.kahraman@uk-essen.de (A.K.); julia.kaelsch@uk-essen.de (J.K.); hc.hsinchiang88@gmail.com (H.C.); guido.gerken@helios-gesundheit.de (G.G.)

**Keywords:** moderate alcohol consumption, NASH, liver enzymes, lipid metabolism, microbiota

## Abstract

Alcoholic liver disease (ALD) and non-alcoholic fatty liver disease (NAFLD) have emerged as leading causes of chronic liver diseases worldwide. ALD and NAFLD share several pathophysiological patterns as well as histological features, while clinically, they are distinguished by the amount of alcohol consumed daily. However, NAFLD coexists with moderate alcohol consumption in a growing proportion of the population. Here, we investigated the effects of moderate alcohol consumption on liver injury, lipid metabolism, and gut microbiota in 30 NAFLD-patients. We anonymously assessed drinking habits, applying the AUDIT- and CAGE-questionnaires and compared subgroups of abstainers vs. low to harmful alcohol consumers (AUDIT) and Cage 0–1 vs. Cage 2–4. Patients who did not drink any alcohol had lower levels of γGT, ALT, triglycerides, and total cholesterol. While the abundance of *Bacteroidaceae*, *Bifidobacteriaceae*, *Streptococcaceae*, and *Ruminococcaceae* was higher in the low to harmful alcohol drinking cohort, the abundance of *Rikenellaceae* was higher in the abstainers. Our study suggests that even moderate alcohol consumption has an impact on the liver and lipid metabolism, as well as on the composition of gut microbiota.

## 1. Introduction

Due to the dramatic increase [1] in the prevalence of obesity and metabolic syndromes over the past decades, non-alcoholic fatty liver disease (NAFLD) has emerged as one of the leading causes of chronic liver diseases worldwide [2]. Establishing an NAFLD diagnosis requires the exclusion of alternate causes of hepatic fat accumulation, including excessive alcohol consumption. With regard to the latter, the European Association for the Study of the Liver (EASL) [3], the American Association for the Study of the Liver (AASLD) [4], and the Asian Pacific Association for the Study of the Liver (APASL) have defined safety levels of drinking, which also serve as a dose threshold to discriminate NAFLD from alcoholic liver disease (ALD) [5]. A simplified definition and diagnosis as well as a renaming to MAFLD (metabolic dysfunction associated fatty liver disease), which is based on changes associated with obesity, is currently under discussion [6]. This definition aims to reduce the stigmatization of NAFLD patients, point out the effects of metabolism on liver injury, and avoid the exclusion of secondary causes.

As ALD is a major cause of chronic liver diseases and given the fact that humans are diverse in habits and diet, it is not surprising that NAFLD coexists with alcohol consumption in a growing proportion of the population. Unfortunately, alcohol-related damage of the liver increases sensitivity for the harmful effects of obesity and metabolic syndromes and vice versa [7].

While ALD and NAFLD demonstrate some similarities in their pathophysiology, both disease entities are initiated by distinct mechanisms. In ALD, hepatic fat accumulation is mainly due to a change in the lipid metabolism, which is primarily based on a change in the redox state of the hepatocyte. Hereby, ethanol oxidation leads to increased triglyceride synthesis and decreased ß-oxidation of fatty acids [8]. In NAFLD, hyperinsulinemia and high circulating levels of free fatty acids are the main factors for fat accumulation in the liver [9]. Hepatic lipid accumulation promotes a cellular increase of reactive oxygen species (ROS), mitochondria and endoplasmic reticulum stress, and increased lipotoxicity [10,11]. NASH (non-alcoholic steatohepatitis) is the progressive form of NAFLD with inflammation and ballooning of hepatocytes and is associated with hepatic and extrahepatic morbidity and mortality [12,13].

The aim of the present study was to investigate the effects of moderate alcohol consumption (MAC) in patients with NASH, and analyze the effects on liver injury, parameters of lipid metabolism and the cytokeratin 18 filament as a biomarker for apoptosis (M30) and overall cell death (M65). As an increasing number of studies have revealed an association between the intestinal microbiota and NAFLD as well as ALD; we additionally aimed to examine the impact of MAC on the composition of the microbiota in patients with NASH in the context of MAC.

## 2. Patients and Methods

### 2.1. Ethics Statement, Patients’ Recruitment, and Acquisition of Data

The study protocol conformed to the ethical guidelines of the 1975 Declaration of Helsinki. All subjects provided informed written consent and the study was approved by the Essen University Hospital Ethics committee (Institutional Review Board; 15-6356-BO). Patients were prospectively recruited in the Department of Gastroenterology and Hepatology at Essen University Hospital from September 2016 until December 2017. Patients diagnosed with NAFLD were prospectively recruited for the study. The single patients were blinded by means of a numerical code. The data obtained were recorded anonymously by commonly used questionnaires, the Alcohol Use Disorders Identification Test (AUDIT; www.auditscreen.org and a German language version, www.bundesaerztekammer.de (accessed on 31 August 2016)), the CAGE questions (cut-down, annoyed, guilty, and eye-opener), and the serum tests, so that the patient provides honest information about his alcohol consumption and the treating physician cannot access this information.

### 2.2. Measurement of Serum Parameters and Urine EtG

Standard laboratory parameters were evaluated via the central laboratory of the Essen University Hospital. Ethylglucoronide (EtG) measurement was taken in the laboratory medicine Eberhard (Dortmund, Germany) in urine samples.

### 2.3. ELISAs

Serum levels of the overall cell death marker M65 and apoptosis marker M30 were quantified using commercially available kits from TecoMedical (Sissach, Switzerland).

### 2.4. Microbiota Analysis

Patients’ fecal samples were collected in sterile tubes and stored at −80 °C until further processing. The isolation of bacterial DNA was performed using the QIAmp DNA isolation kit following the manufacturer’s instructions (Qiagen, Hilden, Germany). For better cell disruption, we used a mechanical lysis step using the Fast Prep™-24 instrument (MP Biomedicas, Solon, OH, USA) at 6.0 m/s for 2 × 45 seconds. The preparation of amplicon libraries was performed as described previously and sequenced on a MiSeq (2 × 250 bp, Illumina, Hayward, CA, USA) [10]. The resulting FastaQ files were analyzed using the dada2 package in R (www.r-project.org (version 3.4.2. 2017)). All samples were resampled to equal the smallest library size of 12,448 reads using the phyloseq package and returning 6341 phylotypes. Sequence reads were assigned to a taxonomic affiliation based on the naïve Bayesian classification with a pseudo-bootstrap threshold of 80%. Relative abundances in percentage of phylogenetic ranks, Phylotypes, Family, Order, and Phylum, were used for further analyses. The abundances of those phylotypes with a mean > 1% were compared by the Mann–Whitney test using GraphPad Prism 9. Detected phylotypes were taxonomically assigned to 16 Phyla, 53 Orders, and 77 Families.

### 2.5. Statistical Analysis

Statistical significance was determined using one-way ANOVA and nonparametric tests (Kruskal–Wallis and Dunn’s multiple comparison test) if not stated otherwise. All analyses were performed with GraphPad Prism 9. If not stated otherwise, all data are presented as mean ± SEM and statistical significance was assumed at *p* ≤ 0.05.

## 3. Results

### 3.1. Patient Demographics and Assessment of Alcohol Consumption

In 2016, 30 patients (14 males and 16 females with a mean age of 56.53 ± 2.44 years), who fulfilled the criteria of NASH according to the current EASL guidelines [14] were asked to evaluate their current drinking habits and the ones two years ago. For the purpose of standardization, we used the AUDIT questionnaire and the CAGE questions for alcohol use and divided the cohort into two major categories, with two subgroups each—abstainers/low to harmful (AUDIT) and Cage 0–1/Cage 2–4 (CAGE) [15].

The study participants of the AUDIT cohort consist of 11 abstainers, who drink no alcohol or up to one time per month and 19 low to harmful alcohol drinkers, with a minimum of 2–4 times and more of alcohol drinking per month. The abstainers’ cohort was comprised of eight females and three males with a mean age of 58.13 years and a mean BMI of 27.9 kg/m^2^. Of the cohort, 54.5% were diabetics and 63.6% were suffering from arterial hypertension. In the low to harmful group, there were eight females and 11 males with a mean age of 57.17 and a mean BMI of 27.73 kg/m^2^. Here, 15.8% were diagnosed with diabetes and 57.9% with arterial hypertension (Table 1).

According to the CAGE questionnaire, we additionally divided the group into two subgroups. The cohort of CAGE 0–1 included 10 females and six males with a mean age of 54.29 years and a mean BMI of 29.79 kg/m^2^. In this subgroup 43.8% were diabetics and 50% had arterial hypertension. The subgroup of the CAGE 2–4 individuals comprises six females and eight males with a mean age of 57.79 and a mean BMI of 27.35kg/m^2^. Within this group, 14.3% suffered from diabetes and 71.4% from arterial hypertension (Table 2).

### 3.2. Liver Injury and Alcohol Consumption in NAFLD

In addition to self-evaluation, we measured the alcohol metabolite EtG, which can be detected in the urine for several days after alcohol consumption. No measurable EtG levels were found in the entire cohort, except for one subject.

To analyze the impact of drinking habits, we examined the current liver parameters (γGT, ALT, AST, and alkaline phosphatase), the parameters of the lipid metabolism (triglycerides, total cholesterol, LDL-, and HDL cholesterol) and the biomarkers M30 (apoptosis) and M65 (cell death) of each patient and assigned the results to the corresponding subgroups.

**Figure 1:**
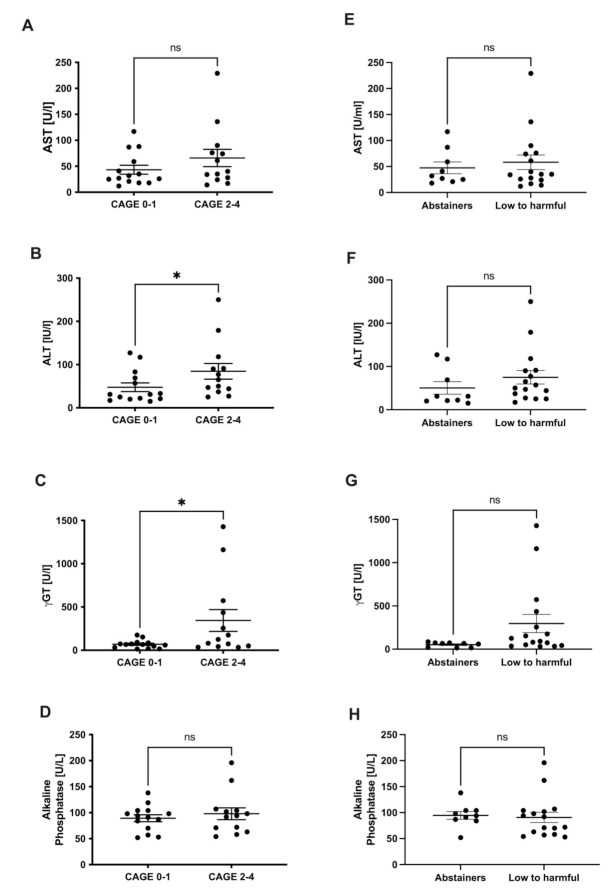
Liver injury parameters. Comparison of parameters of liver injury in serum of NASH patients. Levels of AST (**A**,**E**), ALT (**B**,**F**), γGT (**C**,**G**), and alkaline phosphatase (**D**,**H**) were compared in CAGE 0–1 versus CAGE 2–4 (CAGE questionnaire) and abstainers versus low to harmful consumers (AUDIT questionnaire). * *p* < 0.05. ns stands for not significant.

The patients, who did not consume any alcohol or were categorized as Cage 0–1, had lower levels for AST, ALT, and γGT, whereas the changes for ALT and γGT were significant (Figure 1A–C). Levels for alkaline phosphatase did not show any differences (Figure 1D). A similar pattern was observed for parameters comparing the groups of abstainers and low to harmful consumers (following the AUDIT questionnaire), but the results did not reach statistical significance (Figure 1E–H). We also investigated the impact on liver damage via the biomarkers M30 (apoptosis) and M65 (cell death) and found no significant differences according to both the CAGE and AUDIT categorization (Supplementary Figure S1).

### 3.3. Lipid Metabolism and Alcohol Consumption in NAFLD

**Figure 2:**
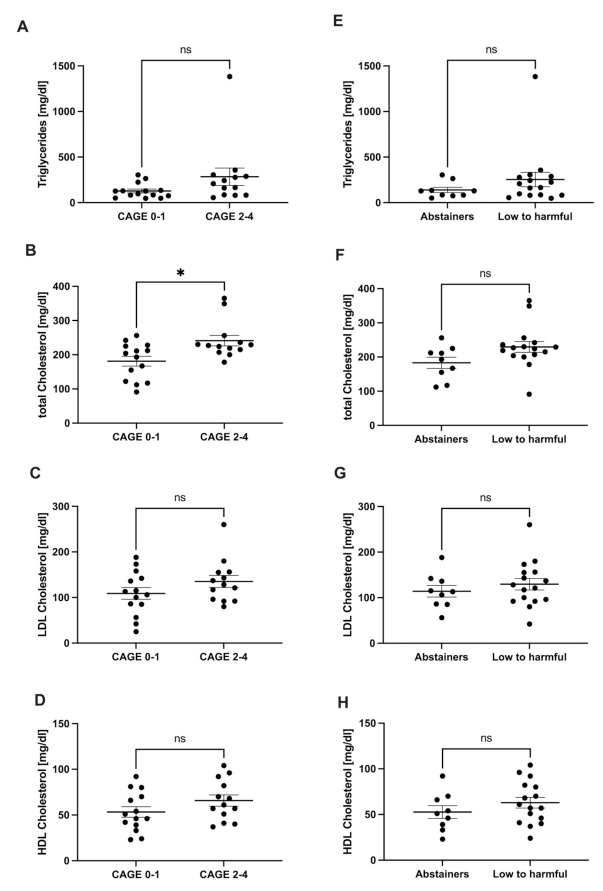
Lipid metabolism. Comparison of serum parameters of lipid metabolism in NASH patients according to drinking history. Levels of triglycerides (**A**,**E**), total cholesterol (**B**,**F**), LDL cholesterol (**C**,**G**), and HDL cholesterol (**D**,**H**) in CAGE 0–1 versus CAGE 2–4 (CAGE questionnaire) and abstainers versus low to harmful consumers (AUDIT questionnaire). * *p* < 0.05. ns stands for not significant.

We quantified serum triglycerides and total cholesterol, as well as HDL- and LDL cholesterol. Individuals that did not consume any alcohol (CAGE 0–1 and abstainers) had lower levels of triglycerides, total cholesterol, LDL cholesterol, and HDL cholesterol, compared to those who did consume alcohol (CAGE2–4 and low to harmful; Figure 2).

### 3.4. Gut Microbiota Composition and Alcohol Consumption in NAFLD

Finally, we analyzed the composition of the gut microbiota comparing two subgroups (abstainers (*n* = 14) vs. low to harmful (*n* = 9), AUDIT questionnaire). The reduced group size is explained by missing fecal samples.

**Figure 3:**
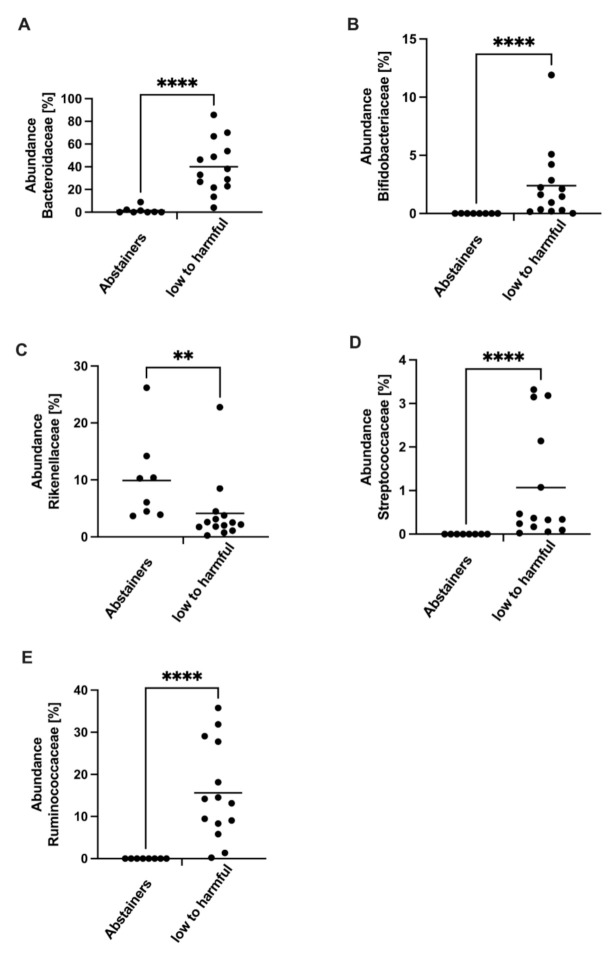
Analysis of microbiota on family taxonomy level. Microbiota analysis comparing relative abundances of bacterial groups on family taxonomy level comparing the composition of *Bacteroidaceae* (**A**), *Bifidobacteriaceae* (**B**), *Rikenellaceae* (**C**), *Streptococcaceae* (**D**), and *Ruminococcaeae* (**E**) in abstainers versus low to harmful consumers (AUDIT questionnaire). ** *p* < 0.01; **** *p* < 0.0001.

We analyzed the composition of the gut microbiota and found significant differences in the abundance of bacterial strains at the family level. While the abundance of *Bacteroidaceae*, *Bifidobacteroidaceae*, *Streptococcaceae*, and *Ruminococcaceae* was decreased in abstainers, the abundance of *Rikenellaceae* was higher in abstainers (Figure 3).

### 3.5. Analysis of Drinking History

Concerning the drinking history as assessed by a two-year recall of the AUDIT questionnaire, most of the abstainers remained abstainers and those who had consumed alcohol two years ago, continued to drink alcohol. Only three patients quitted drinking and no study participants picked up during the two years period. The quitters consisted of three males and the exact reasons for abstinence are not further known. The characteristics of the participants who stopped drinking within 2 years are listed in Supplementary Table S1.

**Figure 4:**
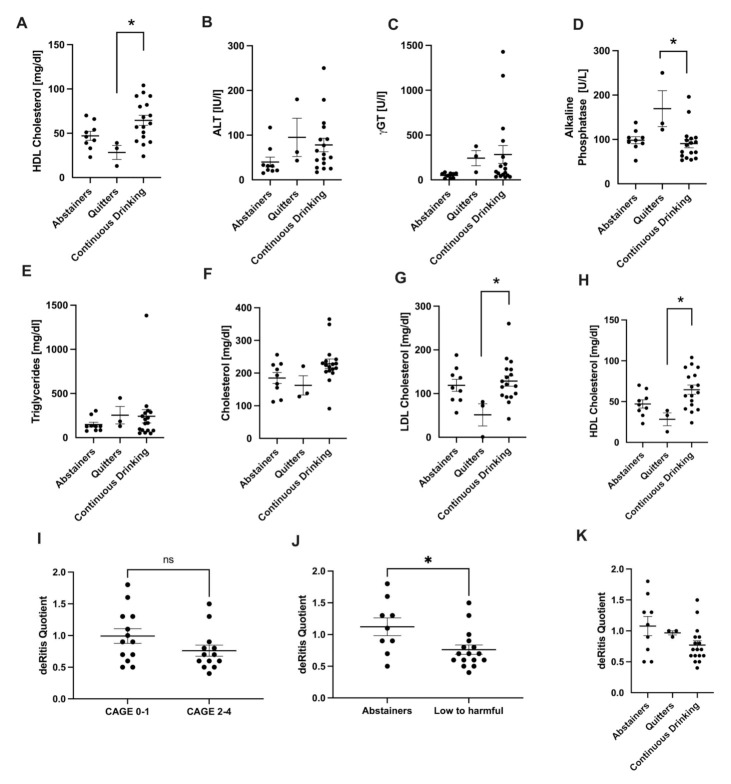
Analysis of drinking history. Serum parameters of liver injury (**A**–**D**) and lipid metabolism (**E**–**H**) were analyzed in abstainers, quitters, and continuously drinking patients. DeRitis quotient was compared in CAGE 0–1 versus CAGE 2–4 ((**I**), CAGE questionnaire), abstainers versus low to harmful consumers ((**J**), AUDIT questionnaire) as well as with regard to the drinking history (**K**). * *p* < 0.05. ns stands for not significant.

Comparing the three groups’ transaminases levels, γGT and alkaline phosphatase tended to be higher in the quitters (Figure 4A–D). Triglycerides and total cholesterol were not changed, while LDL- and HDL-cholesterol were lower in quitters compared to abstainers and continuously drinking individuals. (Figure 4A–H).

Furthermore, we analyzed the impact on the AST/ALT ratio (DeRitis quotient), which is frequently utilized in ALD patients. We found slightly decreased levels in the subgroup with a problematic drinking behavior (CAGE 2–4). Higher levels were observed in individuals that did not drink any alcohol according to the AUDIT questionnaire (Figure 4I,J, *p* < 0.05). There were no significant differences comparing the DeRitis quotient in abstainers, quitters, and continuously drinking patients (Figure 4K).

## 4. Discussion

NAFLD is the leading chronic liver disease worldwide with an estimated global prevalence of 25%. In approximately 10–20% of cases, this can lead to NASH [16]. This subgroup has an increased risk for disease progression, including the development of liver cirrhosis, hepatocellular carcinoma, and/or the need for liver transplantation. Although excessive alcohol intake is an established risk factor for liver disease, it remains disputed to which extent MAC plays a role concerning liver-related complications in NAFLD and NASH.

Defining the alcohol consumption threshold for hepatotoxity itself remains complicated, as it depends on cofactors that may modify the susceptibility of the liver (for example sex, ethnicity, and genetic factors). EASL and the AASLD propose 30g/day and 20g/day of alcohol in men and women, respectively [4]. The APASL is more conservative and proposes 20g/day in men and 10g/day in women [5]. Before examining the effects of MAC in NAFLD, it is also crucial to exclude alternate reasons for a fatty liver disease, such as chronic HCV infection, endocrine disorders, or nutritional/intestinal-related causes [17].

In our study, which included only patients with NASH based on primary NAFLD, we firstly analyzed the impact of the drinking habits on γGT and the transaminases ALT and AST. The patients, who did not consume any alcohol or were categorized as Cage 0–1, had lower levels for AST, ALT, and γGT, whereas alterations in ALT and γGT were significant. Alkaline phosphatase did not show any differences. The clearest differences were found in γGT levels, which confirms the role of γGT as a surrogate marker for relevant alcohol consumption, although other factors, such as obesity, may contribute to an elevation. Additionally, we analyzed the impact on the AST/ALT ratio (DeRitis quotient). AST/ALT was slightly decreased in individuals with problematic drinking behavior (CAGE 2–4). Higher levels were observed in the patients who did not drink any alcohol according to the AUDIT questionnaire. At first glance, this might be surprising, because an increased AST/ALT ratio was historically thought to be specific for excessive alcohol consumption. Most likely, the small number of patients could be a reason for the fact that such established parameters are not applied here in a meaningful way. Recently, this ratio has been suggested to be also indicative for advanced fibrosis [2]. In our study, we did not perform liver biopsy but measured transient elastography (Fibroscan), including the controlled attenuation parameter (CAP), as non-invasive markers of fibrosis and hepatic steatosis. In the measurements, the dataset was unfortunately not complete, however, the available data also demonstrated no statistically significant differences comparing the groups. In a cohort study, Chang et al. demonstrated that MAC was significantly and independently associated with the worsening of non-invasive indices of fibrosis. In their study, they used the NAFLD fibrosis score (NFS) and the Fibrosis-4 Index (FIB-4) [18]. Of course, this would not explain our results, as the AST/ALT ratio was higher in the groups who did not demonstrate a problematic drinking behavior. However, before deriving conclusions, it would be necessary to apply additional non-invasive fibrosis markers at baseline and after two years in both cohorts.

We also investigated the impact on liver damage via the biomarkers M30 and M65. While levels of M30 are considered relatively specific for hepatocellular apoptotic cell death, M65 levels reflect total cell death. In our study, we found no significant differences according to both groupings of CAGE and AUDIT. The results are probably due to the small number of patients and the fact that other contributing factors were not considered [19]. Further studies with larger cohorts should be conducted to evaluate the impact on liver damage applying those biomarkers. CAGE and AUDIT questionnaires were designed to address different aspects of alcohol abuse and raising awareness in different population groups [15]. Therefore, different group sizes result from the utilization of two distinct questionnaires. However, in the clear cohort, there is a clear overlap within the groups. Therefore, the results show the same trends for both group divisions.

Although both ALD and NAFLD inarguably have disease specific features, they mechanistically share common features of pathophysiology. The genetic background of patients plays a crucial role in terms of the severity and progression of both entities and can therefore be considered as disease modifiers [20]. In recent years, several studies have shown that several common genetic variants may influence both ALD and NAFLD, due to the central role of lipid metabolism. That applies, for example, to a common variant (I148M) in the patatin-like phospholipase domain-containing protein 3 (PNPLA3) gene that encodes a protein involved in the lipid metabolism [21], which influences the severity of steatosis, steatohepatitis, fibrosis, and HCC risk in NAFLD as well as in ALD [22]. Similarly, genetic studies have demonstrated that a variant in the transmembrane 6 superfamily member 2 ((TM6SF2) gene is not only associated with an increased risk of liver disease in ALD [23]. It can also contribute to a fat accumulation in the liver due to a disorder of the VLDL-secretion. Interestingly, the same variant seems to be protective against cardiovascular disease [24]. More recently, a variant (rs641738) in the membrane bound O-acyltransferase domain-containing 7-membrane channel-like 4 (MBOAT7) gene has also been demonstrated to have an impact on disease progression in both NAFLD and ALD [23]. It is therefore not unlikely that the consumption of alcohol that influences the course of ALD would also influence the course of NAFLD and the progressive form, NASH, respectively.

Regarding the lipid metabolism, the study participants who did not consume any alcohol (CAGE 0–1 and abstainers), had lower levels of triglycerides, cholesterol, HDL, and LDL, compared to those who did consume any alcohol. A statistical significance was mostly not reached. This could be explained by the small number of patients in the study. In addition to that, other factors such as physical exercise, eating habits, and genetic modifiers, as discussed above, have an impact on the lipid metabolism. Hence, those factors should be taken into consideration when drawing further conclusions. Concerning the eating habits, for example, epidemiological and experimental studies have demonstrated that both the amount and the type of dietary fat have an impact on the pathogenesis of ALD- and NAFLD-related complications [25,26].

Finally, we analyzed the composition of the gut microbiota, whereby significant differences in the abundance of bacterial strains were detected only at the family level. While the abundance of *Bacteroidaceae*, *Bifidobacteroidaceae*, *Streptococcaceae*, and *Ruminococcaceae* was decreased in the abstainers, the abundance of *Rikenellaceae* was higher in the subgroup that was classified as low to harmful.

Several studies have demonstrated that alcoholic liver disease may lead to alterations in gut microbiota composition [27]. It has been demonstrated that microbiota abundance and distribution differ depending on the severity of the disease. Microbiota composition, as well, has an impact on the outcome of therapeutic interventions [28,29]. According to Hartmann et al., differences in individual bacterial species are described in both animal models and patient studies. Comparing different studies reveals shifts in specific species, but the findings frequently remain controversial. In alcoholics with or without cirrhosis, equal shifts of *Bacteriodaceae* were described, but interestingly, the specific abundances in our cohort are reversed [30,31]. In this context, it needs to be considered that these studies included individuals with cirrhosis and/or significant harmful alcohol consumption. In our study, we were concerned with the effect of low levels of alcohol on the progression of NAFLD. We also did not observe a general alcohol-associated dysbiosis, such as SIBO, in our patients.

It is widely accepted that the composition of the gut microbiota has an impact, not only on the carbohydrate and the lipid metabolism, but also on the balance between pro-inflammatory and anti-inflammatory mediators in the liver. An increased gut permeability leads to an enhanced translocation of bacterial endotoxins that are, for example, derived from gram-negative bacteria. After entering the portal circulation, those endotoxins can provoke inflammatory processes in pre-existing NAFLD and ALD [24]. Another contributing factor could be derived from the fact that alcohol is constantly produced by intestinal microbiota in the human gut [16]. Recent studies suggest that a dysbiosis in the intestine could lead to an increased endogenous production of ethanol, which can also trigger inflammation in the liver [32]. What should also be mentioned in this context is that bacterial metabolism and potentially metabolites produced by appropriately established bacterial groups may also have an influence on the host and its energy metabolism. Various representatives of gram-positive bacterial groups, such as *Bifidobacteriaceae* or *Streptococcaceae*, which were increased in the low to harmful alcohol group, influence the regulation of beta-glucoronidase or beta-galctosidase, both of which also have an influence on general metabolic processes in glucose and fat metabolism [29]. Bacterial metabolites, such as short chain fatty acids (SCFA), play a crucial role in the integrity of the intestinal mucosal barrier function and have an influence on systemic and hepatic inflammation, as well as the hepatic energy metabolism [33,34]. *Ruminococcus,* belonging to the family of *Ruminocaccaceae,* have been associated with the organic metabolite Trimethylamine N-oxide (TMAO) levels, which can be associated with an increased cardiovascular risk and higher mortality [35,36]. In our study, in the group of low to harmful drinkers, there were increased abundances of the groups *Bifidobacteriaceae*, *Streptococcaceae,* and *Ruminococcaceae*, indicating an altered energy metabolism and thus a risk of increased hepatic steatosis due to the formation of corresponding bacterial metabolites.

Therefore, it is likely that alterations in the microbiota composition can also lead to a progression of a steatohepatitis, no matter if it is based on NAFLD or ALD. Conversely, recent research focused on the potential and the feasibility of applying the intestinal microbiota as a therapeutic target for NAFLD/NASH [37]. Different concepts, such as the modulation of the microbiota composition by antibiotics, probiotics, or prebiotics have been tried [38]. While it is not surprising that in our study the microbiota composition differs in the two subgroups, it remains unclear whether this is only due to the difference in the amount of alcohol they were consuming. The diet of the subgroups, for example, which certainly has an impact on the bacterial strains, is one of the possible confounding factors, which cannot be excluded. It should also be mentioned that a large proportion of the abstainers were diabetics compared to those who had a higher alcohol consumption. We did not take this fact into account for the more detailed evaluation, as no meaningful comparison could be made due to the small group size. This aspect should also be considered for future studies, as the presence of diabetes can certainly influence corresponding measurement parameters of this study, such as hepatic steatosis, but also the composition of the microbiome. However, it is likely that the consumption of alcohol has an impact on the microbiota and therefore also indirectly on the inflammatory processes in the liver. This opens a new window for future studies. Most certainly we need much larger cohorts, because a lot of observations in this field of research demonstrate their weakness in a lack of reproducibility.

When discussing the association between NAFLD and alcohol, it needs to be clarified on which aspect you are focusing on: the effects of alcohol on the prevalence or incidence of NAFLD, or the effects of alcohol on the course of a pre-existing NAFLD, including the risk of developing a HCC or the impact on mortality in patients with NAFLD [39]. Especially the latter aspect can be controversial, as MAC has been demonstrated to have beneficial effects on cardiovascular mortality in a subset of patients [40].

In a study from the UK, Inan-Enoglu et al. examined the joint associations of adiposity, BMI and waist circumference (WC), and alcohol consumption on ALD, NAFLD, liver disease incidence, and mortality. Alcohol consumption was categorized based on current UK guidelines (14 units/week). They found out that being overweight (BMI)/obese (WC) amplified the harmful effect of alcohol on the liver incidence and mortality [41].

Given the increasing number of patients presenting clinical features of both alcoholic- and metabolic-associated fatty liver disease, it is going to be necessary to develop therapeutic targets. In an experimental approach, Benedé-Ubieto et al. have developed a preclinical DUAL model in mice (alcohol-associated liver disease plus MAFLD), which mimics all histological, metabolic, and transcriptomic gene signatures of human advanced steatohepatitis. Hence, it could serve as a preclinical tool for the development of therapeutic approaches [42].

In summary, our study was limited by the small number of participants and the fact that other contributing factors, such as eating habits, body workout, and genetic modifiers were not taken into consideration. Unfortunately, due to the anonymous data evaluation using the various questionnaires, we did not have access to potentially available patho-histological biopsy reports or the opportunity to take biopsies to correlate the measured parameters with these findings. Our data suggest that even a moderate consumption of alcohol has an impact not only on the liver and the lipid metabolism, but also on the composition of the microbiota. However, we strongly recommend further ideally prospective studies with a higher number of study participants and including other contributing factors, such as eating habits, physical exercise, and genetic preconditions to confirm our data.

## Figures and Tables

**Table 1:** Grouping based on the current drinking habits following statements of the AUDIT questionnaire comparing abstainers (none up to 1× alcohol per month) versus low to harmful drinking (minimum 2–4 × alcohol per month and more).

	Abstainers *n* = 11	Low to Harmful *n* = 19
Age	58.13 ± 5.48	57.17 ± 2.67
BMI	27.90 ± 1.32	27.73 ± 0.74
Sex	8 female/3 male	8 female/11 male
Diabetes	6 (54.5%)	3 (15.8%)
Arterial hypertension	7 (63.6%)	11 (57.9%)

**Table 2:** Comparison based on the CAGE questionnaire comparing CAGE 0–1 point (no risk for alcoholism) versus CAGE 2–4 points (risk for alcoholism).

	CAGE 0–1 *n* = 16	CAGE 2–4 *n* = 14
Age	54.29 ± 3.29	57.79 ± 3.42
BMI	29.79 ± 1.09	27.35 ± 0.91
Sex	10 female/6 male	6 female/8 male
Diabetes	7 (43.8%)	2 (14.3%)
Arterial hypertension	8 (50.0%)	10 (71.4%)

## Data Availability

The data presented in this study are available on request from the corresponding author. The data are not publicly available due to privacy and ethical reasons.

## Supplementary Materials

The following supporting information can be downloaded at: https://www.mdpi.com/article/10.3390/jcm11030890/s1, Figure S1: Cell death parameters. Comparison of hepatocellular apoptosis (M30; A/C) and total cell death (M65, B/D) in serum of NASH patients in CAGE 0–1 versus CAGE 2–4 (following CAGE questionnaire) and abstainers versus low to harmful consumers (following AUDIT questionnaire); Table S1: Characteristics of the Quitters.

## Abbreviations

AASLD: The American association for the study of the liver; ALD: alcoholic liver disease; ALT: alanine aminotransferase; APASL: The Asian Pacific association for the study of the liver; AST: aspartate aminotransferase; AUDIT: alcohol use disorder identification test; BMI: body mass index; CAGE: cut-down, annoyed, guilty, and eye-opener; CAP: controlled attenuation parameter; EASL: European association for the study of the liver; EtG: ethylglucoronide; FIB-4: Fibrosis-4 score; γGT: gamma glutamyl transferase; HDL: high density lipoprotein; LDL: low density lipoprotein; MAC: moderate alcohol consumption; MBOAT7: membrane-bound o-acyltransferase domain-containing 7-membrane channel-like 4; NAFLD: non-alcoholic fatty liver disease; NASH: non-alcoholic steatohepatitis; NFS: NAFLD-fibrosis score; PNPLA3: patatin-like phospholipase domain-containing protein 3; SCFA: short chain fatty acids; SIBO: small intestinal bacterial overgrowth; TMAO: trimethylamine N-oxide; TM6SF2: transmembrane 6 superfamily member 2; VLDL: very low density lipoprotein; WC: waist circumference.
