# Supplementary material for: Effects of Moderate Alcohol Consumption in Non-Alcoholic Fatty Liver Disease
Source: J Clin Med. 2022 Feb 8;11(3):890. doi: 10.3390/jcm11030890 (PMC8836912; doi:10.3390/jcm11030890)
Supplement: Supplementary file 1 [file jcm-11-00890-s001.zip › jcm-1554909-supplementary.pdf]

Supplementary material to the article

Effects of moderate alcohol consumption in non-alcoholic fatty liver disease

Peter Lemmer[1,2], Paul Manka[1], Jan Best[1], Alisan Kahraman[3], Julia Kälsch[3], Ramiro Vilchez-Vargas[2], Alexander Link[2], Hsin Chian[3], Guido Gerken[3], Ali Canbay[1], Lars P. Bechmann[1] and Svenja Sydor[1]

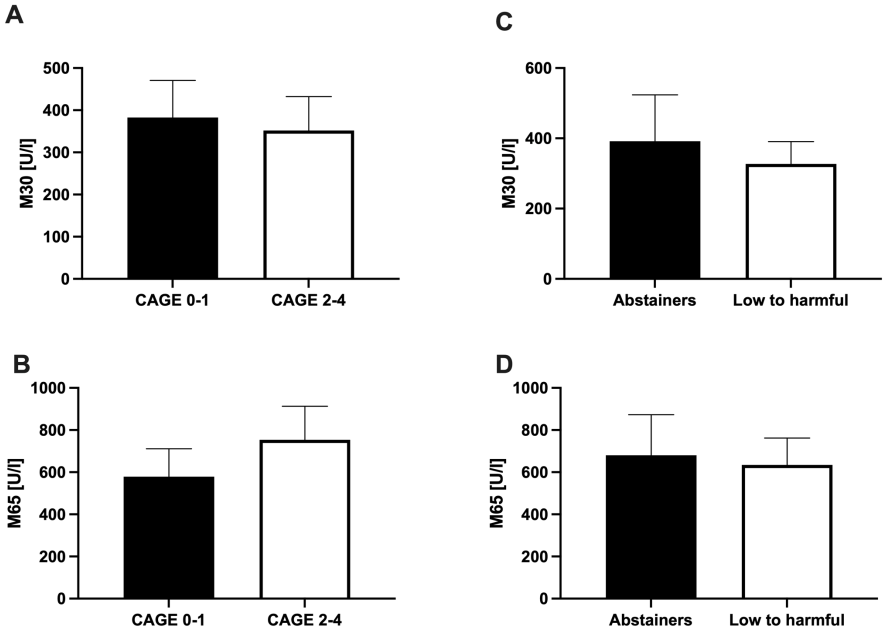

Figure S1. Cell death parameters. Comparison of hepatocellular apoptosis (M30; A/C) and total cell death (M65, B/D) in serum of NASH patients in CAGE 0-1 versus CAGE 2-4 (following CAGE questionnaire) and abstainers versus low to harmful consumers (following AUDIT questionnaire).

Table S1. Characteristics of the Quitters

	**Sex**	**Age**	**BMI**	**Diabetes**	**Arterial hypertension**	**Dyslipidemia**
**Quitter 1**	male	50	29.4	no	yes	yes
**Quitter 2**	male	65	28.7	yes	yes	no
**Quitter 3**	male	55	30.4	no	yes	no
